# Beginning of the Pandemic: COVID-19-Elicited Anxiety as a Predictor of Working Memory Performance

**DOI:** 10.3389/fpsyg.2020.576466

**Published:** 2020-11-26

**Authors:** Daniel Fellman, Liisa Ritakallio, Otto Waris, Jussi Jylkkä, Matti Laine

**Affiliations:** ^1^Department of Applied Educational Science, Umeå University, Umeå, Sweden; ^2^Department of Psychology, Åbo Akademi University, Turku, Finland; ^3^Department of Clinical Neuroscience, Karolinska Institute, Stockholm, Sweden; ^4^Department of Child Psychiatry, University of Turku and Turku University Hospital, Turku, Finland; ^5^INVEST Research Flagship Center, University of Turku, Turku, Finland; ^6^Turku Brain and Mind Center, Turku, Finland

**Keywords:** COVID-19, working memory, anxiety, state anxiety, trait anxiety

## Abstract

Increasing evidence indicates that the coronavirus disease 2019 (COVID-19) pandemic is associated with adverse psychological effects, including heightened levels of anxiety. This study examined whether COVID-19-related anxiety levels during the early stage of the pandemic predicted demanding working memory (WM) updating performance. Altogether, 201 healthy adults (age range, 18–50) mostly from North America and the British Isles were recruited to this study via the crowdsourcing site www.prolific.co. The results showed that higher levels of COVID-19-related anxiety during the first weeks of the pandemic outbreak were associated with poorer WM performance as measured by the n-back paradigm. Critically, the unique role of COVID-19-related anxiety on WM could not be explained by demographic factors, or other psychological factors such as state and trait anxiety or fluid intelligence. Moreover, across three assessment points spanning 5–6 weeks, COVID-19-related anxiety levels tended to decrease over time. This pattern of results may reflect an initial psychological “shock wave” of the pandemic, the cognitive effects of which may linger for some time, albeit the initial anxiety associated with the pandemic would change with habituation and increasing information. Our results contribute to the understanding of cognitive–affective reactions to a major disaster.

## Introduction

The recent coronavirus disease 2019 (COVID-19) pandemic has profoundly altered the lives of countless people. Most countries reacted to the outbreak with recommendations of social distancing (Gharebaghi et al., 2020), and some countries even enacted total self-quarantine (Qiu et al., 2020). Thus, in addition to direct illness-related effects, the COVID-19 pandemic affected most aspects of everyday life in the form of limited freedom of movement (Khoo and Lantos, 2020), increased isolation (Hellewell et al., 2020), and a risk or realized layoff or unemployment (Kawohl and Nordt, 2020). Although preventive actions and medical treatments undoubtedly have the highest priority during the outbreak, the mental consequences of the pandemic on the population are also pivotal. Preliminary reports point to a substantial psychological impact of the pandemic, affecting both healthcare personnel and the general public. These first studies indicate elevated risks of acute stress disorders (Huang et al., 2020), psychosis (Brown et al., 2020), schizophrenia (Hu et al., 2020), and mental illness in general (Li et al., 2020; Liu et al., 2020) as a consequence of the pandemic.

One affect that is particularly relevant during disasters such as COVID-19 is *anxiety*. Natural disasters (e.g., earthquakes, tsunamis, and epidemics) lead to increased levels of anxiety (Dewaraja et al., 2006; Jones and Salathé, 2009; Nakayachi et al., 2015; Hossain et al., 2020), and the COVID-19 pandemic is no exception (Duan and Zhu, 2020; Li et al., 2020; Ozamiz-Etxebarria et al., 2020; Wang et al., 2020). Elevated levels of anxiety during the COVID-19 pandemic can lead to more persistent worry about everyday things (Huang et al., 2020), and evidence from other natural disasters indicate that anxiety is interlinked with other mental health problems, such as posttraumatic stress disorders (Kar and Bastia, 2006) and depression (Angola and Costello, 1993), thereby contributing negatively to the public health crisis. Anxiety can also deteriorate certain aspects of cognitive performance (for a review, see Robinson et al., 2013), and thus, the possible cognitive effects of COVID-19-evoked anxiety should also come under scrutiny.

This study investigated whether and how COVID-19-elicited anxiety is associated with a core cognitive function, working memory (WM), during the early stage of the disease outbreak. WM can be defined as our mental workspace, which is responsible for temporarily maintaining and manipulating information before it decays (Baddeley and Hitch, 1974; Baddeley, 2000, 2003; Cowan et al., 2005). WM is of critical importance for managing tasks that require volitional processing in everyday life such as decision making (Hinson et al., 2003), cognitive control (Poole and Kane, 2009), and understanding false beliefs (Keenan, 1998; Keenan et al., 1998). WM ability could even affect individuals’ tendency to rely on misinformation (Brydges et al., 2018), and WM has been shown to bear direct relevance for the current COVID-19 pandemic. A recent study demonstrated that individuals with poorer WM performance were more prone to disregard social-distancing recommendations during the initial outbreak (Xie et al., 2020). Importantly, a large body of evidence indicates that individual differences in WM are related to anxiety levels so that those with higher anxiety levels tend to show poorer WM performance (Ashcraft and Kirk, 2001; Johnson and Gronlund, 2009; Andreotti et al., 2013; Bredemeier and Berenbaum, 2013). A recent meta-analysis by Moran (2016) verified the outcomes from single studies, demonstrating that anxiety shows a reliable negative association with WM (Hedges’ *g* values from −0.334 to −0.437). While many theories have been put forth to explain the relationship between anxiety and WM, most of them agree on the assumption that the limited capacity of WM is disrupted by “task-irrelevant” worry, which results in poorer cognitive performance (Sarason, 1988; Calvo and Eysenck, 1992; Eysenck et al., 2007). What remains unstudied is how WM is related to anxiety elicited by the exceptional pandemic caused by COVID-19 during the early outbreak.

The present study was conducted with 201 healthy younger adults who were recruited from the online crowdworking platform Prolific Academic^1^. The data was collected within the first 4 weeks (March 18 to April 07, 2020) after the World Health Organization (WHO) had declared COVID-19 a pandemic. COVID-19-induced anxiety was assessed at two different time points during the early outbreak (between March 18 and April 01, 2020 and between March 23 and April 07, 2020) and at a follow-up time point 1 month later (between April 14 and May 1, 2020) using a single item on a continuous Likert scale that asked participants to estimate how anxious they were due to the current COVID-19 pandemic. Besides the COVID-19 anxiety probe, the first assessment point consisted of questionnaires tapping personality features (Openness, Conscientiousness, and Trait anxiety) and state anxiety, whereas the second assessment point consisted of several WM tasks. We administered in total nine WM tasks, consisting of three n-back tasks (the current element has to be matched with the element presented n trials ago; Kirchner, 1958), two running memory tasks (a sequence of items with a random length is presented after which the four last items has to be recalled; Pollack et al., 1959), two simple span tasks (a sequence of items with varying lengths has to be recalled in a serial order; Wechsler, 1997), and two selective updating tasks (a row of items is presented and two of the items are selectively updated with new items; Murty et al., 2011). In the present study, we will focus mainly on the n-back tasks, as this demanding WM task paradigm has been shown to be particularly sensitive to the disruptive effects of anxiety (Bredemeier and Berenbaum, 2013; Balderston et al., 2016; Lukasik et al., 2019).

On the basis of previous evidence regarding anxiety–WM relationships (Moran, 2016), we hypothesized that n-back performance would be negatively associated with COVID-19-elicited anxiety at both COVID-19 anxiety assessment points so that those with higher anxiety levels would have poorer n-back performance during the early outbreak of the pandemic. We were particularly interested to see whether COVID-19 anxiety would be predictive of n-back performance above and beyond demographic factors (age, gender, and education), psychological factors (state and trait anxiety, Big Five Openness to experience, and Conscientiousness), and fluid intelligence. The reason for controlling for the personality factors Openness and Conscientiousness was that previous evidence has linked both of them to WM as measured by n-back (Waris et al., 2018). Reasoning ability was also critical to control for since it is a distinct construct that is highly intercorrelated with WM (Engle et al., 1999; Conway et al., 2003). These two constructs have been suggested to share about 50% of their variance (Ackerman et al., 2005; Kane et al., 2005; Oberauer et al., 2005).

## Materials and Methods

### Participants

Data in this within-subjects study stems from two sequential prescreening assessments, a baseline session, and a follow-up assessment of a WM training study. In that study, the main objective was to elucidate whether a highly varied WM training regime would elicit more flexible strategy use and thereby yield larger generalization effects as compared with repetitive WM training consisting of a single training task, but results pertaining to this research question will be reported elsewhere. The participants were 18–50-year-old healthy adults recruited between March 18 and April 01, 2020, through the crowdworking site Prolific Academic^1^. The study was approved by the Ethics Board of the Departments of Psychology and Logopedics, Åbo Akademi University, and it was conducted in accordance with the Helsinki Declaration. Informed consent was obtained from all participants. The inclusion criteria were as follows: English native speakers, no current psychiatric or neurological illnesses that affected the participant’s daily life, no current use of central nervous system (CNS) medication, and no current psychotropic drug use (except tobacco, alcohol, and cannabis). Altogether, 216 participants completed our two prescreening assessments and the baseline session (for more detailed information, see *Procedure*). For identifying those that had been cheating in the cognitive assessment (administered during the baseline session), participants were asked whether they used any external tools (for example, writing, taking notes, or drawing) to help them solve the tasks after they had completed all the tasks. The participants could respond either “Yes” or “No.” We stressed that the participant’s honest response was critically important and that their response to this question would have no negative consequences for them. In total, 15 participants reported that they had been using external tools, and they were therefore excluded. After excluding those participants, the final sample size was 201. Their average age was 32.13 years (*SD* = 8.25), average education length was 16.13 years (*SD* = 3.34), and 57.43% were female (*n* = 112). Most of the participants resided in the United Kingdom (*n* = 131; 65.2%) or the United States (*n* = 50; 24.9%), whereas the rest resided in Canada (*n* = 7; 3.5%), Australia (*n* = 8; 4.0%), and Ireland (*n* = 5; 2.5%).

### Procedure

**FIGURE 1:**
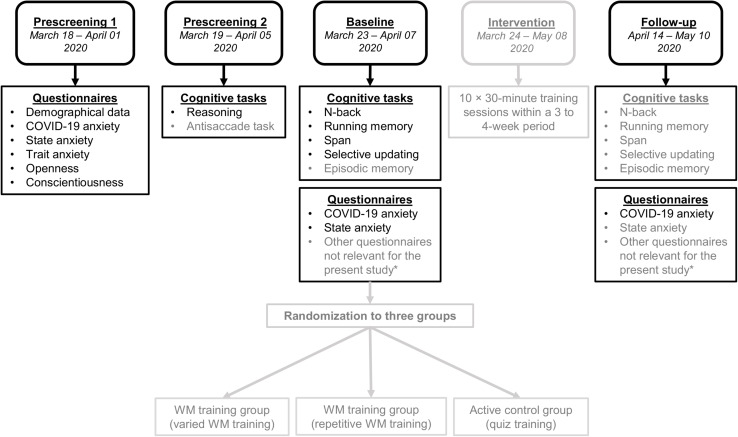
The five main stages of the study design. Those assessment points and measures not used in the present study are shaded in gray. *Other questionnaires implemented at baseline and/or follow-up included a questionnaire capturing subjective WM functioning in daily activities and questionnaires on the use of external or internal memory aids and strategies. We also surveyed engagement (i.e., motivation and alertness) and whether the participant was intoxicated during cognitive task performance.

These data stem from a WM training study that included five stages: prescreening round 1, prescreening round 2, baseline assessment (i.e., pretest), intervention, and posttest (see also Figure 1 for a summary of the five main stages in the study). In the first prescreening round, the participants answered questions about their background (e.g., age, gender, and health), personality, state/trait anxiety, and the single question on COVID-19-induced anxiety. Those participants that were English native speakers in the age range of 18–50 years who had no current psychiatric or neurological illnesses and reported no current use of CNS medication or psychotropic drugs (except tobacco, alcohol, and cannabis) were further invited to the second prescreening round. In the second prescreening round, the participants completed two cognitive measures: one reasoning task (ICAR-16) and one inhibition task (an antisaccade task that is not reported in the present study). Besides gathering information about the participants’ reasoning and inhibition abilities, these measures in the second prescreening also served to detect unreliable effort, which is a common concern in online experiments (e.g., Ford, 2017). In this study, unreliable effort was defined as being three times the interquartile range below the first quartile in the reasoning task. No participant performed below this threshold. During the baseline assessment that was administered between March 23 and April 07, 2020, the participants completed nine WM tasks (see *Materials* for more detailed descriptions), two episodic memory tasks (these tasks will not be reported in more detail in this study), and were asked to respond to the State anxiety questionnaire and the COVID-19 anxiety item once again. Following baseline, the participants were randomized into three interventions: either to one of two groups receiving two different variants of WM training or an active control group training with quiz tasks tapping general knowledge (for more information of the training regimes, see our preregistered study protocol at^2^). Following the intervention phase, all participants took part in a final follow-up assessment. The follow-up assessment was administered between April 14 and May 1, 2020; and it encompassed the same nine WM tasks as in the baseline assessment as well as a third iteration of the COVID-19 anxiety item.

**FIGURE 2:**
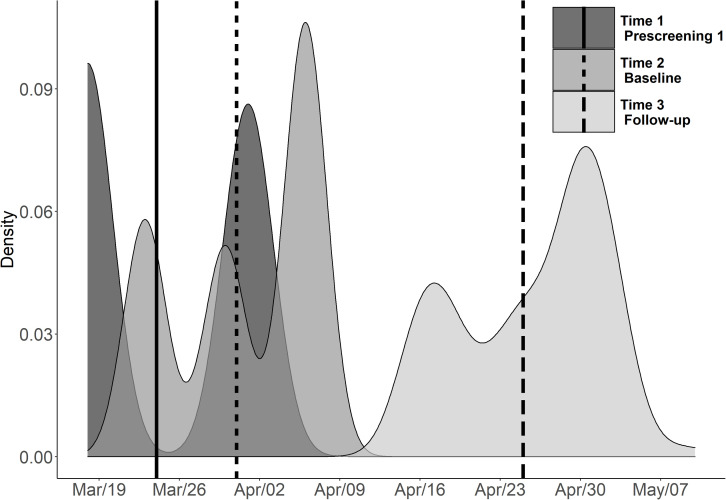
Depiction of the average time point and density plots for the COVID-19 anxiety item responses grouped by assessment point in Spring 2020. The solid vertical line represents the average timepoint for the first assessment (Time 1/Prescreening 1), the dashed vertical line represents the average time point for the second assessment (Time 2/Baseline), and the vertical long dash line represents the average time point for the third assessment (Time 3/Follow-up). Note that the questionnaires tapping on personality were administered during the prescreening, whereas the WM assessment was carried out during the baseline assessment.

Density plots and the average time point for each of the three COVID-19 anxiety assessments can be found in Figure 2. The mean time point for the first COVID-19 anxiety assessment was March 24; for the second assessment, March 31; and for the third assessment, April 25. The participants received £0.68 (approximately $0.83) for prescreening round 1, £2.34 (approximately $2.85) for prescreening round 2, and £47.50 (approximately $57.89) for completing the sequence of baseline assessment, intervention, and the follow-up assessment.

### Materials

#### COVID-19 Anxiety

This item asked participants to estimate how anxious they were about the current COVID-19 pandemic. The endpoints of the scale were defined as follows: “Not at all, it does not worry me the slightest” = 1; “Crippling, constant worry that interferes with daily activities and thinking, possibly including, for example, panic attacks and/or severe and frequent restlessness = 10.”

#### Demographics

We collected information on the participants’ age and gender. Moreover, they were asked to report the length of their education in years.

#### State Anxiety

For measuring State anxiety, we administered a short form of the state scale of the Spielberger State Trait Anxiety Inventory (STAI-6) developed by Marteau and Bekker (1992). The participants reported their current general level of anxiety according to six statements (e.g., “I am worried”) on a 4-point Likert scale (“not at all” = 1, to “very much” = 4). Possible scores range from 6 to 24, with high scores indicating high levels of state anxiety. Cronbach’s alpha for State anxiety during time 1 and 2 was 0.84, and 0.85, respectively, indicating a good level of internal consistency.

#### Personality

Three personality features were assessed, namely, Openness to experience, Conscientiousness, and Trait anxiety. Openness and Conscientiousness were assessed with the Big-Five Inventory 2 (BFI-2) questionnaire (Soto and John, 2017). Cronbach’s alpha was 0.87 for the Conscientiousness subscale and 0.85 for the Openness subscale, indicating good levels of internal consistency. Trait anxiety was measured using the subscale from the International Personality Item Pool-HEXACO domain (Ashton et al., 2007). It contains altogether 10 items (e.g., “Get stressed out easily,” “Worry about things”). Possible scores on this scale range from 1 to 50, with higher scores indicating higher levels of trait anxiety. Cronbach’s alpha was 0.92, indicating good internal consistency.

#### Reasoning

For measuring individual differences in reasoning, the participants completed the 16-item International Cognitive Ability Resource measure (ICAR-16; Condon and Revelle, 2014). It consists of 16 items separated into four item types (with four items per type): (1) Matrix reasoning, (2) Letter and number series, (3) Verbal reasoning, and (4) Three-dimensional rotation. The participants received 1 point for each correctly solved item (i.e., score range, 0–16) and had unlimited time to complete the task. In this study, Cronbach’s alpha for the ICAR-16 was 0.78, indicating good internal consistency.

#### Working Memory

Our WM assessment battery comprised nine WM tasks, and the present data from these tasks stem from the baseline assessment prior to the intervention period. The tasks consisted of three adaptive n-back tasks, two simple span tasks with fixed sequences, two running memory tasks with fixed sequences, and two selective updating tasks with fixed sequences. For the present study, we focused mainly on the n-back tasks, as previous evidence indicates that this demanding WM task paradigm is particularly sensitive to the disruptive effects of anxiety (Bredemeier and Berenbaum, 2013; Balderston et al., 2016; Lukasik et al., 2019). The n-back tasks were otherwise identical to each other but differed with respect to stimuli that were either digits (1, 2, 3, 4, 5, 6, 7, 8, and 9), letters (A, B, C, D, E, F, G, H, and I), or colors (blue, yellow, red, green, purple, black, pink, orange, and gray). The items in each n-back task were presented one at a time on a computer screen, and the participants were instructed to respond “yes” or “no” to each item with a computer keyboard press, indicating whether the current item corresponded to the item presented n items back in the sequence. Each task variant consisted of 12 blocks, with each block containing 20 + *n* trials. Out of the 20 trials in a block, 6 were targets and 14 non-targets. Four of the non-targets were lures (i.e., identical to the target items except that they were presented *n* ± 1 back), which were meant to increase the task demands and discourage familiarity-based responding. Stimulus display time for each item in a sequence was 1,500 ms, whereas the interstimulus interval was 450 ms. The n-back tasks were adaptive so that task difficulty depended on the participant’s success rate. Each n-back task started with a 1-back block, and the level of *n* could vary between 1 and 12. If the participant recalled 18–20 trials correctly in a block, the program increased the level of *n* by one. The level of *n* remained the same if the participant recalled 15–17 trials correctly, while 5 or more incorrectly recalled trials resulted in a decrease of *n* by one. As the three n-back measures correlated quite strongly with each other (*r*s > 0.63), we created a composite WM variable, consisting of a z-transformed performance score from baseline of the mean n-back level from each n-back variant, which were then averaged together.^1^ The test descriptions pertaining to the Running memory, Simple span, and Selective updating paradigms are summarized in the Supplementary materials (Appendix A).

### Analytical Approach

All statistical analyses were conducted using the R version 3.5.2 (R Core Team, 2016). First, to examine whether the COVID-19-related anxiety changed over time, we computed a repeated measures ANOVA where COVID-19 anxiety served as the dependent variable and Time (prescreening, baseline, and follow-up) as the within-subjects variable. Second, the association between COVID-19 anxiety and n-back was assessed using a hierarchical multiple linear regression analysis. The baseline n-back composite score served as the dependent variable in the analyses. At step 1, we entered the demographic control variables age, gender, and education (in years) together with the personality variables Openness, Conscientiousness, and Trait anxiety and the ICAR-16 reasoning measure. At step 2, we entered the State anxiety variable. Lastly, at step 3, we entered the COVID-19 anxiety variable that was the predictor of interest. As we sought to examine whether COVID-19-elicited anxiety and its relationship to WM would be stable across time during the early outbreak, we computed two separate hierarchical multiple regression models. These models where otherwise identical, differing only with respect to the COVID-19 item of interest. Specifically, the first model encompassed the COVID-19 anxiety from the first prescreening round, whereas the second model encompassed the COVID-19 anxiety item from the baseline assessment. Moreover, as the anxiety measures at time 1 and time 2 (i.e., State anxiety and COVID-19 anxiety) were temporally overlapping and close to each other in time (see Figure 2), we also ran an additional analysis where we averaged the State anxiety scores and the COVID-19 anxiety scores across the two assessment points. These measures were fed into a third hierarchical multiple regression model. Lastly, given that all participants underwent cognitive interventions following baseline that most likely impacted their n-back performance, we considered it methodologically inappropriate to analyze the cognitive data from the follow-up assessment point in a predictive regression model.

## Results

We screened the WM composite variable for univariate outliers. Those who scored three times the interquartile range above or below the first or third quartile in the composite WM score were defined as outliers. However, no such outliers were detected, allowing us to include all participants in the analyses. Table 1 depicts descriptive statistics for the variables. Table 2 lists the zero-order correlations between the WM composite and the predictors. These correlations showed a statistically significant negative association between n-back performance at the second assessment point and COVID-19 anxiety both during the first assessment point (i.e., prescreening 1) (*r* = −0.180, *p* = 0.011) and about a week later during the second assessment point (i.e., baseline assessment) (*r* = −0.178, *p* = 0.011).

**TABLE 1:** Descriptive statistics of the test variables.

					**Range**	
**Variable**	***M***	***SD***	**Skew**	**Kurtosis**	**Actual**	**Potential**
Conscientiousness	44.57	8.44	–0.23	–0.54	23–60	12–60
Openness	44.95	8.16	–0.41	–0.05	18–60	12–60
Trait anxiety	29.81	8.98	–0.03	–0.61	10–50	10–50
Reasoning^a^	7.73	3.61	0.18	–0.58	0–16	0–16
State anxiety time 1	11.47	3.76	0.66	0.25	6–24	6–24
State anxiety time 2	10.89	3.57	0.85	0.94	6–24	6–24
State anxiety (mean)	11.18	3.32	0.75	0.85	6–24	6–24
COVID-19 anxiety time 1	5.62	1.97	–0.29	–0.75	1–10	1–10
COVID-19 anxiety time 2	5.53	1.96	–0.34	–0.64	1–10	1–10
COVID-19 anxiety (mean)	5.58	1.84	–0.29	–0.55	1–10	1–10
COVID-19 anxiety time 3	5.01	1.96	–0.14	–0.89	1–10	1–10

*Descriptives from the demographical variables, which can be found in *Methods*, are excluded from the table. State anxiety (mean) consists of the averaged score from State anxiety time 1 and State anxiety time 2. COVID-19 anxiety (mean) consists of the averaged score from COVID-19 anxiety time 1 and COVID-19 anxiety time 2. 1 = prescreening 1; time 2 = baseline; 3 = follow-up. ^*a*^Stems from the second prescreening round.*

**TABLE 2:** Correlation matrix between the background variables and test variables.

**Variable**	**1**	**2**	**3**	**4**	**5**	**6**	**7**	**8**	**9**	**10**	**11**	**12**	**13**	**14**	**15**
1. Age	–														
2. Gender	–0.09	–													
3. Education	0.16*	0	–												
4. Openness	0.04	0.05	0.21**	–											
5. Conscientiousness	0.20**	–0.05	0.05	0.13	–										
6. Trait anxiety	−0.14*	−0.29**	−0.19**	–0.13	−0.18*	–									
7. Reasoning^a^	0.08	0.09	0.13	0.09	−0.25**	0.02	–								
8. State anxiety time 1	0.01	−0.16*	−0.18*	–0.09	–0.14	0.63**	0.01	–							
9. State anxiety time 2	0.06	–0.13	–0.07	–0.08	−0.16*	0.52**	0.05	0.64**	–						
10. State anxiety (mean)	0.04	−0.16*	−0.14*	–0.09	−0.16*	0.64**	0.03	0.91**	0.90**	–					
11. COVID-19 anxiety time 1	0.11	−0.15*	–0.09	0.07	0.04	0.30**	0.01	0.43**	0.43**	0.47**	–				
12. COVID-19 anxiety time 2	0.09	−0.16*	–0.09	0.07	0.06	0.29**	–0.09	0.33**	0.37**	0.38**	0.74**	–			
13. COVID-19 anxiety (mean)	0.1	−0.17*	–0.1	0.08	0.05	0.31**	–0.04	0.41**	0.43**	0.46**	0.93**	0.93**	–		
14. COVID-19 anxiety time 3	0.07	–0.07	–0.06	0.04	0.03	0.24**	–0.06	0.35**	0.39**	0.41**	0.63**	0.66**	0.69**	–	
15. WM (n-back) time 2	–0.07	0.07	0.12	0.07	−0.17*	–0.03	0.50**	–0.05	0	–0.03	−0.18*	−0.18*	−0.19**	–0.11	–

State anxiety (mean) consists of the averaged score from State anxiety time 1 and State anxiety time 2. COVID-19 anxiety (mean) consists of the averaged score from COVID-19 anxiety time 1 and COVID-19 anxiety time 2. 1 = prescreening 1; time 2 = baseline; 3 = follow-up. *p < 0.05. **p < 0.01. ^*a*^Stems from the second prescreening round.

**FIGURE 3:**
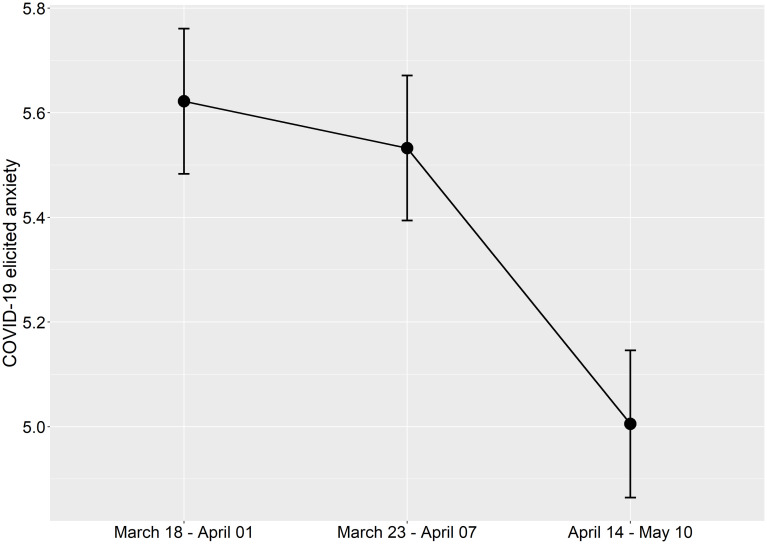
Mean values of COVID-19-related anxiety on a 1–10 scale across the three different assessment points. The error bars represent standard error of means.

We examined whether the COVID-19 anxiety levels changed over time using a repeated measures ANOVA (see Figure 3). The results revealed a main effect of time [*F*(2,582) = 5.488, *p* = 0.004, $\eta_{\text{p}}^{2}$ = 0.019], mainly stemming from the fact that the anxiety scores during the third assessment (i.e., follow-up) were clearly lower than the anxiety levels during the earlier assessment points. Thus, COVID-19-induced anxiety was highest close to the initial outbreak of the pandemic in the West and then decreased during the follow-up.

For summary statistics of the results obtained in the three hierarchical multiple linear regression models, see Tables 3, 4, and 5. When testing the assumptions in the hierarchical multiple linear regression models, the results showed that multicollinearity was not a concern (tolerance range, 0.53–0.91; VIF range, 1.10–1.90). Moreover, an analysis of standard residuals was carried out, showing that the data contained no outliers (standard residual min = −1.86, standard residual max = 2.85). The data also met the assumption of independent errors (Durbin–Watson value = 2.00), and the histogram of standardized residuals revealed that the data comprised approximately normally distributed errors.

**TABLE 3:** Summary of hierarchical regression analysis for variables predicting n-back performance at the first assessment point between March 18 and April 01, 2020.

	**Step 1**				**Step 2**				**Step 3**			
**Predictor**	***B***	***SE B***	**B**	**Sig.**	***B***	***SE B***	**β**	**Sig.**	***B***	***SE B***	**β**	**Sig.**
Age	–0.01	0.01	–0.12	0.078	–0.01	0.01	–0.11	0.095	–0.01	0.01	–0.09	0.158
Gender	0.00	0.12	0.00	0.985	0.00	0.12	0.00	0.998	–0.02	0.12	–0.01	0.852
Education	0.02	0.02	0.07	0.29	0.02	0.02	0.07	0.313	0.01	0.02	0.06	0.388
Openness	0.00	0.01	0.01	0.885	0.00	0.01	0.01	0.88	0.00	0.01	0.03	0.628
Conscientiousness	0.00	0.01	–0.04	0.564	0.00	0.01	–0.04	0.547	0.00	0.01	–0.03	0.677
Trait anxiety	0.00	0.01	–0.05	0.448	0.00	0.01	–0.03	0.729	0.00	0.01	–0.02	0.848
Reasoning	0.12	0.02	0.49	<0.001	0.12	0.02	0.49	<0.001	0.12	0.02	0.49	<0.001
State anxiety time 1					–0.01	0.02	–0.03	0.669	0.01	0.02	0.04	0.678
COVID-19 anxiety time 1									–0.08	0.03	–0.18	0.009
*R*^2^		0.271***				0.272***				0.298***		
*R*^2^ change						0.001				0.026**		

*p < 0.05. **p < 0.01. ***p < 0.001.

**TABLE 4:** Summary of hierarchical regression analysis for variables predicting n-back performance at the second assessment point between March 23 and April 07, 2020.

	**Step 1**				**Step 2**				**Step 3**			
**Predictor**	***B***	***SE B***	**B**	**Sig.**	***B***	***SE B***	**β**	**Sig.**	***B***	***SE B***	**β**	**Sig.**
Age	–0.01	0.01	–0.115	0.078	–0.01	0.01	–0.11	0.095	–0.01	0.01	–0.099	0.133
Gender	0.00	0.12	–0.001	0.985	0.00	0.12	0	0.998	–0.01	0.12	–0.008	0.906
Education	0.02	0.02	0.069	0.29	0.02	0.02	0.066	0.313	0.02	0.02	0.06	0.359
Openness	0.00	0.01	0.009	0.885	0.00	0.01	0.01	0.88	0.00	0.01	0.024	0.711
Conscientiousness	0.00	0.01	–0.038	0.564	0.00	0.01	–0.04	0.547	0.00	0.01	–0.033	0.622
Trait anxiety	0.00	0.01	–0.051	0.448	0.00	0.01	–0.029	0.729	0.00	0.01	–0.012	0.887
Reasoning	0.12	0.02	0.492	<0.001	0.12	0.02	0.492	<0.001	0.12	0.02	0.481	<0.001
State anxiety time 2					–0.01	0.02	–0.034	0.669	0.00	0.02	–0.007	0.933
COVID-19 anxiety time 2									–0.05	0.03	–0.116	0.086
*R*^2^		0.271***				0.272***				0.283***		
*R*^2^ change						0.001				0.011		

*p < 0.05 **p < 0.01 ***p < 0.001

**TABLE 5:** Summary of hierarchical regression analysis for variables predicting n-back performance using the average score of State anxiety (step 2) and COVID-19 anxiety (step 2) across the two assessment points between March 18 and April 07, 2020.

	**Step 1**				**Step 2**				**Step 3**			
**Predictor**	***B***	***SE B***	**B**	**Sig.**	***B***	***SE B***	**β**	**Sig.**	***B***	***SE B***	**β**	**Sig.**
Age	–0.01	0.01	–0.115	0.078	–0.01	0.01	–0.117	0.088	–0.01	0.01	–0.099	0.131
Gender	0.00	0.12	–0.002	0.98	0.00	0.12	–0.002	0.984	–0.03	0.12	–0.015	0.82
Education	0.02	0.02	0.069	0.29	0.02	0.02	0.069	0.295	0.01	0.02	0.056	0.383
Openness	0.00	0.01	0.010	0.881	0.00	0.01	0.010	0.881	0.00	0.01	0.033	0.605
Conscientiousness	0.00	0.01	–0.038	0.564	0.00	0.01	–0.037	0.559	0.00	0.01	–0.024	0.724
Trait anxiety	0.00	0.01	–0.051	0.449	0.00	0.01	–0.060	0.602	0.00	0.01	–0.036	0.672
Reasoning	0.12	0.02	0.492	<0.001	0.12	0.02	0.492	<0.001	0.12	0.02	0.485	<.001
State anxiety (mean)					0.00	0.02	0.016	0.906	0.02	0.02	0.067	0.436
COVID-19 anxiety (mean)									–0.09	0.03	–0.178	0.012
*R*^2^		0.271***				0.271***				0.295***		
*R*^2^ change						<0.001				0.012*		

*p < 0.05 **p < 0.01 ***p < 0.001

The results from the first model (the first assessment point stemming from the first prescreening round) revealed that the first step involving the demographic variables, the three personality variables, and reasoning performance predicted 27.1% of n-back performance *F*(7,193) = 10.26, *p* < 0.001, *R*^2^ = 0.271. Of these predictors, only reasoning was significantly related to n-back performance, such that those performing better in the reasoning task also had higher scores on n-back (β = 0.492, *t*(193) = 7.55, *p* < 0.001). When introducing the State anxiety measure at step 2, the model fit did not increase significantly, Δ*F*(8,192) = 0.189, *p* = 0.66, Δ*R*^2^ = 0.001. However, when the COVID-19 anxiety variable was added to the model in step 3, the model fit improved to a significant degree, Δ*F*(8, 191) = 7.009, *p* = 0.009, Δ*R*^2^ = 0.026). More specifically, those with higher COVID-19 anxiety levels at prescreening tended to have poorer n-back performance at baseline after controlling for demographical characteristics, personality, and general anxiety (β = −0.182, *t*(191) = −2.650, *p* = 0.009).

In the second multiple regression model (see Table 4), the COVID-19 item and the State anxiety variable stemmed from the second assessment point (i.e., baseline assessment). The State anxiety measure introduced at step 2 did not explain any additional variance in *n*-back performance, Δ*F*(8,192) = 0.020, *p* = 0.888, Δ*R*^2^ < 0.001. When adding COVID-19 anxiety to the model in step 3, the results showed a trend for an increase in Δ*R*^2^, Δ*F*(8,191) = 2.986, *p* = 0.086, Δ*R*^2^ = 0.0112). Thus, albeit not reaching statistical significance, those with higher COVID-19-related anxiety levels still tended to have poorer WM *n*-back performance after controlling for demographical characteristics, personality, and general anxiety [β = −0.182, *t*(191) = −1.728, *p* = 0.086].

The output of the third multiple regression model, in which we averaged the scores of State anxiety and COVID-19 anxiety across the two assessment points is summarized in Table 5. The averaged State anxiety measure introduced at step 2 did not significantly explain any additional variance in n-back performance, Δ*F*(8,192) = 0.014, *p* = 0.905, Δ*R*^2^ < 0.001. However, the model fit increased significantly when the averaged COVID-19 anxiety measure was included in step 3, Δ*F*(8,191) = 6.370, *p* = 0.012, Δ*R*^2^ = 0.024).

### Follow-Up Analyses

To elucidate whether the association between WM and COVID-19 anxiety was specific to the n-back paradigm, we conducted follow-up analyses on the three other WM paradigms (i.e., running memory, simple span, selective updating) included in the test battery. All tasks were standardized according to its paradigm in a similar fashion as the n-back tasks. At time point 1, after controlling for step 1 and 2 control variables, COVID-19 anxiety did not predict performance in the Running memory paradigm [Δ*F*(8,190) = 0.792, *p* = 0.378, Δ*R*^2^ = 0.004], the Span paradigm [Δ*F*(8,191) = 0.029, *p* = 0.865, Δ*R*^2^ = <0.001], or the Selective updating paradigm [Δ*F*(8,191) = 0.158, *p* = 0.691, Δ*R*^2^ <0.001]. The same non-significant relationships were repeated at time point two for the Running memory paradigm [Δ*F*(8,190) = 0.781, *p* = 0.378, Δ*R*^2^ = 0.004], the Span paradigm [Δ*F*(8,191) = 0.413, *p* = 0.521, Δ*R*^2^ = 0.002], and the Selective updating paradigm [Δ*F*(8,191) = 0.005, *p* = 0.941, Δ*R*^2^ < 0.001]. More detailed test statistics (including a correlation matrix and coefficients for the hierarchical multiple regression analyses are included in the Supplementary materials (Appendix B). Thus, it appears that the anxiety elicited by the COVID-19 pandemic manifested specifically in the demanding n-back tasks that call for continuous monitoring and updating of information in WM.

## Discussion

There is accumulating evidence of the negative psychological effects related to the COVID-19 pandemic (Brown et al., 2020; Hu et al., 2020; Huang et al., 2020; Li et al., 2020; Liu et al., 2020), including increased levels of anxiety (Duan and Zhu, 2020; Li et al., 2020; Ozamiz-Etxebarria et al., 2020; Wang et al., 2020). In an online follow-up study (*N* = 201), we tested how an important cognitive system, WM, is associated with COVID-19-related anxiety during the early stages of the pandemic in Anglosphere countries. To untangle this association, the participants responded to an item on COVID-19-induced anxiety (assessed between March 18 and April 01, 2020) and responded to the same item again about 1 week (assessed between March 23 and April 07, 2020) and 1 month later (between April 14 and May 1, 2020). During the second assessment point, the participants also completed a set of WM tasks, including three variants of the widely used *n*-back task (Kirchner, 1958). The results showed that COVID-19-elicited anxiety was significantly associated with n-back performance. Critically, at the first assessment, this association held even after controlling for individual differences in demographic factors (age, gender, and education), psychological factors (state and trait anxiety, Big Five Openness to experience, and Conscientiousness), and fluid intelligence. At the second assessment point, the zero-order correlation between COVID-19 anxiety and n-back performance was also significant, even though the unique variance they shared tended to be slightly weaker after adjusting for the aforementioned control variables. However, accumulating support for the significant impact that COVID-19-induced anxiety had on n-back performance stems from a third analysis, in which we averaged the State anxiety and COVID-19 elicited anxiety scores across the two initial assessment points. The results from that analysis showed that the relationship between n-back performance and COVID-19 elicited anxiety remained statistically significant.

Another finding in the present study was that COVID-19-elicited anxiety decreased over time. More specifically, the mean of perceived anxiety due to COVID-19 was highest during the first assessment point (*M* = 5.62), with a slight decrease 1 week later (*M* = 5.53), and a more evident reduction in anxiety levels about 1 month following the first assessment point (*M* = 5.01). These findings stand in contrast to a recent study by Ozamiz-Etxebarria et al. (2020), who assessed Spanish participants with the anxiety questionnaire Depression Anxiety and Stress Scale-21 (DASS) at two time points, namely, the week during which the state of emergency was declared in Spain (between March 11 and March 18) and when people had been in lockdown for about 20 days (between April 2 and April 12). Their results showed that the anxiety levels were higher during the lockdown period as compared to the emergency period, making the authors speculate that the reason lies in the limitations the lockdown imposed on everyday life. On the other hand, DASS is not specifically designed for capturing anxiety due to pandemics, whereas our COVID-19 anxiety item was specifically administered for that purpose. This might thus be one underlying factor in this discrepancy.

It was somewhat surprising that while we found a unique contribution of COVID-19-elicited anxiety on n-back performance, the same relationship was not observed between WM and the two other anxiety measures, especially State anxiety that the COVID-19 items should also reflect (note that State anxiety did show a significant positive correlation with COVID-19 anxiety). This is also discrepant with the recent meta-analysis by Moran (2016) who found that general anxiety shows a reliable negative association with WM. We can only speculate upon the mechanisms that underlie the unique initial contribution of anxiety elicited by COVID-19 on WM that we observed in the present study. One possible explanation is that a threat of a natural disaster provokes more fear, eliciting a stronger disruptive effect on cognition (Helton et al., 2011), whereas the State anxiety measure not explicitly tapping the current major stressor of COVID-19 (albeit decreasing in mean value over the follow-up period, see Table 1) would lead to a more diffuse response that is not solely influenced by the specific current global stressor.

The COVID-19 anxiety items from the first two assessment points correlated equally strongly with WM as measured by n-back (both *r*s = 0.18), but only the COVID-19 anxiety item from the first assessment point significantly increased Δ*R*^2^ in the hierarchical multiple regression model after taking into account variance from our control variables. However, in our third multiple hierarchical regression model, in which we averaged the State anxiety and COVID-19-elicited anxiety scores across the two initial assessment points, the relationship between n-back performance and COVID-19 anxiety remained significant. This result adds to our conclusion that increased COVID-19 anxiety during the initial phase of the pandemic was associated with worse n-back performance. Nevertheless, it is worthwhile to ponder why COVID-19 anxiety from the first assessment point, but not the second, showed a unique statistically significant relationship with n-back performance. Besides error variance that always permeates cognitive assessments, a more theoretical assumption could be that the limited capacity of WM is more disrupted by the initial shock and apprehension of the pandemic threat, leading to more “task-irrelevant” worry (Sarason, 1988; Calvo and Eysenck, 1992; Eysenck et al., 2007) in the beginning of the pandemic. The circa 1 week in between the COVID-19 anxiety assessments may have led to some habituation in the initial affective reaction. In line with this, the levels of COVID-19-induced anxiety were slightly higher during the first assessment point as compared to the second assessment point. At the same time, it is intriguing that the second COVID-19 anxiety assessment point coincides with the WM assessment, meaning that earlier pandemic anxiety was a better predictor of WM than the concomitant one. In other words, the initial emotional reaction to the pandemic appeared to have a somewhat stronger relationship with WM. It is also worth pointing out that the decrease in COVID-19-induced anxiety across time was not unique to this variable. An *a posteriori* repeated measures ANOVA with State anxiety as the dependent variable and Time (prescreening, baseline, follow-up) as the within-subjects variable mimicked the pattern observed for COVID-19 anxiety [*F*(2,582) = 4.994, *p* = 0.007, $\eta_{\text{p}}^{2}$ = 0.017]. This finding seems to suggest that State anxiety also partly captures COVID-19-elicited anxiety, yet in a less specific way.

Another finding that should be noted here is that women tended to show slightly more anxiety as a result of the COVID-19 pandemic as compared with men (*r* = −0.15–−0.16). This is in line with previous evidence both in non-pandemic circumstances (Stumpf et al., 2015), in the context of natural disasters (Lee et al., 2016), as well as in the current COVID-19 pandemic (Huang et al., 2020), where women tend to be more vulnerable to mental health problems. Moreover, we did not find any significant relationship between age or education and COVID-19 anxiety. Here, one could note the results from a recently conducted study that found that education did not predict social distancing in the early COVID-19 outbreak although age did so (Xie et al., 2020).

An interesting finding, revealed in the follow-up analyses (see *Follow-Up Analyses*), is that only n-back performance was related to the COVID-19-elicited anxiety measure, whereas the other WM paradigms (i.e., Running memory, Span, and Selective updating) did not show such an association. There may be several reasons for this. One reason could be that the n-back paradigm is a highly demanding and novel task paradigm for the participants, calling for continuous monitoring and updating of information in WM, whereas the other task paradigms call for more active recall processes (Jaeggi et al., 2010). It is also rather well-established that the n-back correlates only modestly with other WM tasks (for a meta-analytic review, see Redick and Lindsey, 2013), suggesting that it measures somewhat different subcomponents of WM. Studies also show that n-back taps on other cognitive processes than merely WM, such as familiarity- and recognition-based discrimination processes (Kane et al., 2007), inhibition (Kwong See and Ryan, 1995), and cognitive control (Gray et al., 2003). Second, the n-back tasks in the present study were adaptive across 12 blocks, meaning that the performance level was adjusted according to participants’ performance, effectively keeping them at the upper limit of their performance level. This was not the case for the rest of the tasks where the sequences were fixed irrespective of how well the participants performed. Thus, we speculate that the aforementioned specific n-back features, as well as the previously shown relationships between especially *n*-back performances and anxiety (Bredemeier and Berenbaum, 2013; Balderston et al., 2016; Lukasik et al., 2019), are behind the unique relationship between n-back performance and COVID-19 anxiety in the present study.

A research topic directly relevant for the present study is the continued influence effect (CIE), which refers to the tendency to rely on misinformation even after an explicit correction has been provided (Johnson and Seifert, 1994). A study by Brydges et al. (2018) encompassing three factor-analytic experiments showed that WM was significantly related to CIE: those participants with poorer WM performance were more susceptible to believe in misinformation after correcting information had been provided. The possible reason for this could be that limited WM resources prohibit an efficient encoding of the presented information, leading to greater susceptibility to the CIE. From this perspective, our findings raise the question as to whether individuals with higher COVID-19-related anxiety and consequently lower WM processing capacity could be more prone to misinformation which is common in the pandemic (Frenkel et al., 2020; Russonello, 2020). This could impede people from engaging in behaviors that prevent the spread of infection, or conversely, result in overcautious behavior involving utter social isolation.

### Limitations and Conclusion

An issue worth pointing out pertains to the directionality of our results. As the present study was non-experimental, the statistically significant relationship between COVID-19 during the early outbreak and WM could also exist in an opposite direction, namely, that lesser WM capacity makes one more prone to COVID-19-elicited anxiety. An individual’s executive abilities, including WM, play an important role in self-regulation (Hofmann et al., 2012). Another issue pertaining to the generalizability of our results concerns the sample that consisted of relatively young adults (age range, 18–50 years) free from any neurological or mental illnesses. Thus, our sample is not representative of the whole adult population, and it did not include the older age groups that are at particular risk due to COVID-19. A recent report from China indicated that the COVID-19 pandemic elicits distress especially among older adults above 60 years (Qiu et al., 2020), presumably due to the fact that they belong to the age group that have the highest mortality rate (Ozamiz-Etxebarria et al., 2020). Concerning our three assessments of COVID-19 anxiety, it is worth underscoring that there were large overlaps regarding when the participants took the assessments, especially between the first and second assessment point (see Figure 1). This is clearly a limitation; our findings could have been more conclusive if the data from a given assessment point had derived from a narrower time interval. Lastly, COVID-19-elicited anxiety in this study was assessed using a single-item self-report measure, which had not been validated previously. Measuring a construct with a single item poses methodological problems regarding content validity, sensitivity, and reliability (Meyer et al., 1981). On the other hand, previous studies have shown that some constructs can in fact be measured adequately using single items (Scarpello and Campbell, 1983; Nagy, 2002). The construct we aimed to tap (i.e., the degree of COVID-19-related anxiety) was also quite narrow and specific. While we encourage proper validation studies of multi-item disaster-related anxiety questionnaires, one should point out that the particular item we used was primarily designed to measure anxiety during the initial psychological “shock wave” of the pandemic.

Another issue pertaining to our COVID-19 item concerns how it was conceptualized. As previously mentioned, the participants were prompted to “estimate how anxious they were about the current coronavirus (COVID-19) pandemic.” As such, this statement is general and prevents the identification of the specific sources of this anxiety. There could be high interindividual variation regarding the reasons for the anxiety, such as anxiety for becoming infected, some close relatives getting infected, possibly losing job, lack of information on the consequences of the novel pandemic, and so on. The conceptualization of COVID-19 in this study is thus a clear limitation, and this limitation should be kept in mind when considering the present results and conclusions.

The current study reveals an association between COVID-19 anxiety and WM as measured by *n*-back during the early stages of the pandemic. The critical role of WM in anxiety under typical circumstances has previously been established (Moran, 2016), but this finding extends it to the context of natural disasters as well. This contributes to our understanding of individual reactions to major disasters, providing knowledge that is relevant for understanding the current public health crisis.

## Data Availability Statement

The raw data supporting the conclusions of this article will be made available by the authors, without undue reservation.

## Ethics Statement

The studies involving human participants were reviewed and approved by The Institutional Review Board of the Departments of Psychology and Logopedics, Åbo Akademi Ethics Committee. The patients/participants provided their written informed consent to participate in this study.

## Author Contributions

LR, OW, and JJ developed the study concept and all authors contributed to the study design. LR collected the data. DF performed the data analyses and drafted the manuscript. All coauthors provided critical revisions and approved the final version of the manuscript for submission.

## Conflict of Interest

The authors declare that the research was conducted in the absence of any commercial or financial relationships that could be construed as a potential conflict of interest.

## Footnotes

1 www.prolific.co

2 https://osf.io/c9ygt
