# Supplementary material for: Beginning of the Pandemic: COVID-19-Elicited Anxiety as a Predictor of Working Memory Performance
Source: Front Psychol. 2020 Nov 26;11:576466. doi: 10.3389/fpsyg.2020.576466 (PMC7725684; doi:10.3389/fpsyg.2020.576466)
Supplement: Supplementary file 1 [file Table_1.DOCX]

**Appendix A**

*Task descriptions of the Running memory tasks, Forward simple span tasks, and Selective updating tasks*.

**Running memory.** Our task battery included two Running memory tasks where participants were prompted to recall a given number of last items from a suddenly aborted sequence. One of those was a Running memory task with letters (RML) whereas the other one was a Running memory task with colors (RMC). In both variants, stimulus presentation time was 1000 milliseconds and interstimulus interval was 500 ms. In RML, sequences of unrelated letters with unpredictable sequence length were shown on the screen, after which the participant was to recall the last four items in the correct order. The RMC was otherwise identical to the RML, but the stimuli were colors. In both task variants, the participant completed eight trials in a randomized order, with sequence lengths ranging from 4 to 11 items (one trial of each list length). The dependent variable for the Running memory tasks was the number of correct items recalled in correct serial order.

**Span.** We administered two forward simple span tasks, one with letters as stimuli (FSL), and one with colors as stimuli (FSC). In forward simple span tasks, participants are prompted with a sequence of items (presentation time 1000 ms, interstimulus interval 500 ms), and the task is to recall the items in the order in which they were presented. The sequence lengths ranged from 3 to 9 in both variants, and the item sequences were presented in a randomized order. Participants completed one trial of each sequence length. We used the partial credit scoring procedure by tallying the number of correctly recalled items irrespective of list length.

**Selective updating.** The Selective updating (SU) paradigm builds upon the task introduced by Murty et al. (2011). In this study, we administered two selective updating tasks, one with digits (range 1-9) as stimuli (SUD) and one with colors (blue, yellow, red, green, purple, black, pink, orange, and gray) as stimuli (SUC). Otherwise the two selective updating tasks were identical to each other (thus, the word ‘item’ pertains to both variants hereafter in the task description). In both tasks, five unrelated items were presented on the computer screen in a row of five boxes. The participants were instructed to memorize the item sequence. After this, the initial item sequence disappeared, followed by a new row of five boxes. Two of the new boxes contained new items, while three were empty. The participants were prompted to replace the old items with the items presented most recently in the memorized sequence, while maintaining the unchanged items in WM. In both SUD and SUC, participants completed 10 baseline trials (i.e., no updating stages), and 10 trials with three updating stages (i.e., replacement of old items with new ones). At the end of each trial, participants were instructed to report the final item sequence, including the most recent updates. The order of the sequences was randomized for the participants, and they were unaware whether the next sequence would be a baseline sequence or an updating sequence. The initial item sequence was shown for 4000 ms, followed by a 100 ms blank screen, after which the first updating stage was presented for 2000 ms. The updating stage was once again followed by a 100 ms blank screen and the next updating stage. After all the updating stages had been presented (none in the baseline condition), a recall grid with horizontally aligned boxes containing the numbers from 1 to 9 appeared on-screen. The participants were to click on the box numbers in correct order (see Laine, Fellman, Waris, & Nyman, 2018 for more technical details). The dependent variable for initial task performance in SUD and SUC comprised of the average amount of correctly recalled items stemming from the ten updating sequences.

**References**

Laine, M., Fellman, D., Waris, O., & Nyman, T. J. (2018). The early effects of external and internal strategies on working memory updating training. *Scientific Reports*, *8*(1). doi: 10.1038/s41598-018-22396-5

Murty, V. P., Sambataro, F., Radulescu, E., Altamura, M., Iudicello, J., Zoltick, B., … Mattay, V. S. (2011). Selective updating of working memory content modulates meso-cortico-striatal activity. *NeuroImage*, *57*(3), 1264–1272. doi: 10.1016/j.neuroimage.2011.05.006
