# Supplementary material for: Beginning of the Pandemic: COVID-19-Elicited Anxiety as a Predictor of Working Memory Performance
Source: Front Psychol. 2020 Nov 26;11:576466. doi: 10.3389/fpsyg.2020.576466 (PMC7725684; doi:10.3389/fpsyg.2020.576466)
Supplement: Supplementary file 2 [file Table_2.docx]

**Appendix B**

**Correlation matrix**

| **Table B1.** Correlation matrix of the background variables and test variables. | | | | | | | | | | | | | | | | |
| --- | --- | --- | --- | --- | --- | --- | --- | --- | --- | --- | --- | --- | --- | --- | --- | --- |
| Variable | 1 | 2 | 3 | 4 | 5 | 6 | 7 | 8 | 9 | 10 | 11 | 12 | 13 | 14 | 15 | 16 |
| 1. Age | - |  |  |  |  |  |  |  |  |  |  |  |  |  |  |  |
| 2. Gender | -0.09 | - |  |  |  |  |  |  |  |  |  |  |  |  |  |  |
| 3. Education | .16* | 0.00 | - |  |  |  |  |  |  |  |  |  |  |  |  |  |
| 4. Openness | 0.04 | 0.05 | .21** | - |  |  |  |  |  |  |  |  |  |  |  |  |
| 5. Conscientiousness | .20** | -0.05 | 0.05 | 0.13 | - |  |  |  |  |  |  |  |  |  |  |  |
| 6. Trait anxiety | -.14* | -.29** | -.19** | -0.13 | -.18* | - |  |  |  |  |  |  |  |  |  |  |
| 7. Reasoning^a^ | 0.08 | 0.09 | 0.13 | 0.09 | -.25** | 0.02 | - |  |  |  |  |  |  |  |  |  |
| 8. State anxiety time 1 | 0.01 | -.16* | -.18* | -0.09 | -0.14 | .63** | 0.01 | - |  |  |  |  |  |  |  |  |
| 9. State anxiety time 2 | 0.06 | -0.13 | -0.07 | -0.08 | -.16* | .52** | 0.05 | .64** | - |  |  |  |  |  |  |  |
| 10 COVID-19 anxiety time 1 | 0.11 | -.15* | -0.09 | 0.07 | 0.04 | .30** | 0.01 | .43** | .43** | - |  |  |  |  |  |  |
| 11. COVID-19 anxiety time 2 | 0.09 | -.16* | -0.09 | 0.07 | 0.06 | .29** | -0.09 | .33** | .37** | .74** | - |  |  |  |  |  |
| 12. COVID-19 anxiety time 3 | 0.07 | -0.07 | -0.06 | 0.04 | 0.03 | .24** | -0.06 | .35** | .39** | .63** | .66** | - |  |  |  |  |
| 13. Working memory (n-back) | -0.07 | 0.07 | 0.12 | 0.07 | -.17* | -0.03 | .50** | -0.05 | 0.00 | -.18* | -.18* | -0.11 | - |  |  |  |
| 14. Working memory (span) | 0.05 | 0.10 | 0.03 | 0.04 | -0.11 | -.14* | .37** | 0.01 | 0.08 | 0.00 | 0.00 | .15* | .33** | - |  |  |
| 15. Working memory (running memory) | -0.10 | 0.02 | 0.07 | 0.12 | 0.01 | -0.12 | .29** | -0.05 | -0.04 | -0.08 | 0.00 | 0.11 | .33** | .54** | - |  |
| 16. Working memory (selective updating) | -0.08 | 0.01 | .19** | 0.07 | 0.00 | -0.07 | .37** | -0.09 | 0.02 | -0.02 | -0.05 | 0.06 | .46** | .49** | .56** | - |
| Note. * indicates p < .05. ** indicates p < .01.  1 = prescreening 1; time 2 = baseline; 3 = follow-up  ^a^Stems from the second prescreening round. | | | | | | | | | | | | | | | | |

**Hierarchical regression analyses**

| **Table B2** Summary of hierarchical regression analysis for variables predicting WM performance in the Running memory composite score at the first assessment point between March 18 and April 01, 2020. | | | | | | | | | | | | | | |
| --- | --- | --- | --- | --- | --- | --- | --- | --- | --- | --- | --- | --- | --- | --- |
|  | Step 1 | | | |  | Step 2 | | | |  | Step 3 | | | |
|  |  | | | |  |  | | | |  |  | | | |
| Predictor | *B* | *SE B* | β | Sig. |  | *B* | *SE B* | β | Sig. |  | *B* | *SE B* | β | Sig. |
| Age | -0.10 | 0.04 | -0.169 | 0.017 |  | -0.11 | 0.04 | -0.180 | 0.013 |  | -0.10 | 0.04 | -0.173 | 0.017 |
| Gender | -0.66 | 0.72 | -0.066 | 0.355 |  | -0.69 | 0.72 | -0.068 | 0.339 |  | -0.73 | 0.72 | -0.073 | 0.311 |
| Education | 0.03 | 0.10 | 0.017 | 0.814 |  | 0.04 | 0.11 | 0.023 | 0.745 |  | 0.03 | 0.11 | 0.019 | 0.785 |
| Openness | 0.04 | 0.04 | 0.062 | 0.376 |  | 0.04 | 0.04 | 0.061 | 0.387 |  | 0.04 | 0.04 | 0.069 | 0.333 |
| Conscientiousness | 0.05 | 0.04 | 0.084 | 0.25 |  | 0.05 | 0.04 | 0.088 | 0.23 |  | 0.06 | 0.04 | 0.092 | 0.208 |
| Trait anxiety | -0.08 | 0.04 | -0.141 | 0.056 |  | -0.11 | 0.05 | -0.191 | 0.04 |  | -0.10 | 0.05 | -0.186 | 0.046 |
| Reasoning | 0.46 | 0.10 | 0.330 | < .001 |  | 0.46 | 0.10 | 0.331 | < .001 |  | 0.46 | 0.10 | 0.332 | < .001 |
| State anxiety time 1 |  |  |  |  |  | 0.10 | 0.12 | 0.078 | 0.374 |  | 0.14 | 0.12 | 0.104 | 0.263 |
| COVID-19 anxiety time 1 |  |  |  |  |  |  |  |  |  |  | -0.17 | 0.19 | -0.067 | 0.378 |
| *R^2^* |  | 0.138*** |  |  |  |  | 0.141*** |  |  |  |  | 0.145*** |  |  |
| *R^2^* change |  |  |  |  |  |  | .004 |  |  |  |  | .004 |  |  |
| * indicates *p* < .05 ** indicates *p* < .01 *** indicates *p* < .001  Note. There was one participant with missing data in the Running memory task. Thus, the *n* is 198 in the present analyses. | | | | | | | | | | | | | | |

| **Table B3** Summary of hierarchical regression analysis for variables predicting WM performance in the Running memory composite score at the first assessment point between March 18 and April 01, 2020. | | | | | | | | | | | | | | |
| --- | --- | --- | --- | --- | --- | --- | --- | --- | --- | --- | --- | --- | --- | --- |
|  | Step 1 | | | |  | Step 2 | | | |  | Step 3 | | | |
|  |  | | | |  |  | | | |  |  | | | |
| Predictor | *B* | *SE B* | β | Sig. |  | *B* | *SE B* | β | Sig. |  | *B* | *SE B* | β | Sig. |
| Age | -0.10 | 0.04 | -0.169 | 0.017 |  | -0.11 | 0.04 | -0.178 | 0.014 |  | -0.11 | 0.04 | -0.183 | 0.012 |
| Gender | -0.66 | 0.72 | -0.066 | 0.355 |  | -0.68 | 0.72 | -0.068 | 0.342 |  | -0.64 | 0.72 | -0.063 | 0.376 |
| Education | 0.03 | 0.10 | 0.017 | 0.814 |  | 0.02 | 0.10 | 0.016 | 0.819 |  | 0.03 | 0.11 | 0.021 | 0.767 |
| Openness | 0.04 | 0.04 | 0.062 | 0.376 |  | 0.04 | 0.04 | 0.063 | 0.373 |  | 0.03 | 0.04 | 0.054 | 0.443 |
| Conscientiousness | 0.05 | 0.04 | 0.084 | 0.25 |  | 0.05 | 0.04 | 0.089 | 0.227 |  | 0.05 | 0.04 | 0.084 | 0.255 |
| Trait anxiety | -0.08 | 0.04 | -0.141 | 0.056 |  | -0.10 | 0.05 | -0.171 | 0.047 |  | -0.10 | 0.05 | -0.179 | 0.038 |
| Reasoning | 0.46 | 0.10 | 0.330 | < .001 |  | 0.46 | 0.10 | 0.330 | < .001 |  | 0.46 | 0.10 | 0.336 | < .001 |
| State anxiety time 2 |  |  |  |  |  | 0.08 | 0.11 | 0.054 | 0.499 |  | 0.05 | 0.12 | 0.034 | 0.683 |
| COVID-19 anxiety time 2 |  |  |  |  |  |  |  |  |  |  | 0.17 | 0.19 | 0.066 | 0.378 |
| *R^2^* |  | 0.138*** |  |  |  |  | 0.140*** |  |  |  |  | 0.143*** |  |  |
| *R^2^* change |  |  |  |  |  |  | .002 |  |  |  |  | .004 |  |  |
| * indicates *p* < .05 ** indicates *p* < .01 *** indicates *p* < .001  Note. There was one participant with missing data in the Running memory task. Thus, the *n* is 198 in the present analyses. | | | | | | | | | | | | | | |

| **Table B4** Summary of hierarchical regression analysis for variables predicting WM performance in the Forward simple span composite score at the first assessment point between March 18 and April 01, 2020. | | | | | | | | | | | | | | |
| --- | --- | --- | --- | --- | --- | --- | --- | --- | --- | --- | --- | --- | --- | --- |
|  | Step 1 | | | |  | Step 2 | | | |  | Step 3 | | | |
|  |  | | | |  |  | | | |  |  | | | |
| Predictor | *B* | *SE B* | β | Sig. |  | *B* | *SE B* | β | Sig. |  | *B* | *SE B* | β | Sig. |
| Age | 0.00 | 0.01 | 0.021 | 0.765 |  | 0.00 | 0.01 | 0 | 0.999 |  | 0.00 | 0.01 | -0.001 | 0.987 |
| Gender | 0.04 | 0.12 | 0.021 | 0.765 |  | 0.03 | 0.12 | 0.016 | 0.816 |  | 0.03 | 0.12 | 0.017 | 0.807 |
| Education | -0.01 | 0.02 | -0.046 | 0.512 |  | -0.01 | 0.02 | -0.033 | 0.637 |  | -0.01 | 0.02 | -0.032 | 0.646 |
| Openness | 0.00 | 0.01 | -0.002 | 0.973 |  | 0.00 | 0.01 | -0.004 | 0.951 |  | 0.00 | 0.01 | -0.006 | 0.934 |
| Conscientiousness | 0.00 | 0.01 | -0.046 | 0.522 |  | 0.00 | 0.01 | -0.037 | 0.601 |  | 0.00 | 0.01 | -0.038 | 0.595 |
| Trait anxiety | -0.01 | 0.01 | -0.156 | 0.032 |  | -0.03 | 0.01 | -0.257 | 0.005 |  | -0.03 | 0.01 | -0.258 | 0.005 |
| Reasoning | 0.09 | 0.02 | 0.362 | < .001 |  | 0.09 | 0.02 | 0.365 | < .001 |  | 0.09 | 0.02 | 0.364 | < .001 |
| State anxiety time 1 |  |  |  |  |  | 0.04 | 0.02 | 0.160 | 0.063 |  | 0.04 | 0.02 | 0.155 | 0.088 |
| COVID-19 anxiety time 1 |  |  |  |  |  |  |  |  |  |  | 0.01 | 0.03 | 0.013 | 0.865 |
| *R^2^* |  | 0.161*** |  |  |  |  | 0.176*** |  |  |  |  | 0.176*** |  |  |
| *R^2^* change |  |  |  |  |  |  | .015 |  |  |  |  | <.001 |  |  |
| * indicates *p* < .05 ** indicates *p* < .01 *** indicates *p* < .001 | | | | | | | | | | | | | | |

| **Table B5** Summary of hierarchical regression analysis for variables predicting WM performance in the Forward simple span composite score at the second assessment point between March 23 and April 07, 2020. | | | | | | | | | | | | | | |
| --- | --- | --- | --- | --- | --- | --- | --- | --- | --- | --- | --- | --- | --- | --- |
|  | Step 1 | | | |  | Step 2 | | | |  | Step 3 | | | |
|  |  | | | |  |  | | | |  |  | | | |
| Predictor | *B* | *SE B* | β | Sig. |  | *B* | *SE B* | β | Sig. |  | *B* | *SE B* | β | Sig. |
| Age | 0.00 | 0.01 | 0.021 | 0.765 |  | 0.00 | 0.01 | -0.009 | 0.894 |  | 0.00 | 0.01 | -0.013 | 0.853 |
| Gender | 0.04 | 0.12 | 0.021 | 0.765 |  | 0.02 | 0.12 | 0.014 | 0.841 |  | 0.03 | 0.12 | 0.017 | 0.806 |
| Education | -0.01 | 0.02 | -0.046 | 0.512 |  | -0.01 | 0.02 | -0.048 | 0.489 |  | -0.01 | 0.02 | -0.044 | 0.524 |
| Openness | 0.00 | 0.01 | -0.002 | 0.973 |  | 0.00 | 0.01 | 0.000 | 0.997 |  | 0.00 | 0.01 | -0.006 | 0.932 |
| Conscientiousness | 0.00 | 0.01 | -0.046 | 0.522 |  | 0.00 | 0.01 | -0.028 | 0.698 |  | 0.00 | 0.01 | -0.031 | 0.661 |
| Trait anxiety | -0.01 | 0.01 | -0.156 | 0.032 |  | -0.03 | 0.01 | -0.262 | 0.002 |  | -0.03 | 0.01 | -0.268 | 0.002 |
| Reasoning | 0.09 | 0.02 | 0.362 | < .001 |  | 0.09 | 0.02 | 0.362 | < .001 |  | 0.09 | 0.02 | 0.366 | < .001 |
| State anxiety time 2 |  |  |  |  |  | 0.05 | 0.02 | 0.196 | 0.012 |  | 0.04 | 0.02 | 0.182 | 0.026 |
| COVID-19 anxiety time 2 |  |  |  |  |  |  |  |  |  |  | 0.02 | 0.03 | 0.047 | 0.521 |
| *R^2^* |  | 0.161*** |  |  |  |  | 0.188*** |  |  |  |  | 0.190*** |  |  |
| *R^2^* change |  |  |  |  |  |  | .027* |  |  |  |  | .002 |  |  |
| * indicates *p* < .05 ** indicates *p* < .01 *** indicates *p* < .001 | | | | | | | | | | | | | | |

| **Table B6** Summary of hierarchical regression analysis for variables predicting WM performance in the Selective updating composite score at the first assessment point between March 14 and April 01, 2020. | | | | | | | | | | | | | | |
| --- | --- | --- | --- | --- | --- | --- | --- | --- | --- | --- | --- | --- | --- | --- |
|  | Step 1 | | | |  | Step 2 | | | |  | Step 3 | | | |
|  |  | | | |  |  | | | |  |  | | | |
| Predictor | *B* | *SE B* | β | Sig. |  | *B* | *SE B* | β | Sig. |  | *B* | *SE B* | β | Sig. |
| Age | -0.02 | 0.01 | -0.172 | 0.012 |  | -0.02 | 0.01 | -0.170 | 0.015 |  | -0.02 | 0.01 | -0.172 | 0.014 |
| Gender | -0.10 | 0.12 | -0.059 | 0.389 |  | -0.10 | 0.12 | -0.059 | 0.396 |  | -0.10 | 0.12 | -0.057 | 0.414 |
| Education | 0.04 | 0.02 | 0.146 | 0.033 |  | 0.04 | 0.02 | 0.144 | 0.037 |  | 0.04 | 0.02 | 0.146 | 0.035 |
| Openness | 0.00 | 0.01 | -0.011 | 0.873 |  | 0.00 | 0.01 | -0.010 | 0.877 |  | 0.00 | 0.01 | -0.014 | 0.839 |
| Conscientiousness | 0.01 | 0.01 | 0.113 | 0.107 |  | 0.01 | 0.01 | 0.112 | 0.113 |  | 0.01 | 0.01 | 0.110 | 0.121 |
| Trait anxiety | -0.01 | 0.01 | -0.079 | 0.265 |  | -0.01 | 0.01 | -0.065 | 0.468 |  | -0.01 | 0.01 | -0.067 | 0.455 |
| Reasoning | -0.02 | 0.01 | -0.172 | 0.012 |  | 0.10 | 0.02 | 0.404 | < .001 |  | 0.10 | 0.02 | 0.404 | < .001 |
| State anxiety time 1 |  |  |  |  |  | 0.00 | 0.02 | -0.023 | 0.788 |  | -0.01 | 0.02 | -0.034 | 0.705 |
| COVID-19 anxiety time 1 |  |  |  |  |  |  |  |  |  |  | 0.01 | 0.03 | 0.029 | 0.691 |
| *R^2^* |  | 0.197*** |  |  |  |  | 0.197*** |  |  |  |  | 0.198*** |  |  |
| *R^2^* change |  |  |  |  |  |  | <.001 |  |  |  |  | <.001 |  |  |
| * indicates *p* < .05 ** indicates *p* < .01 *** indicates *p* < .001 | | | | | | | | | | | | | | |

| **Table B7** Summary of hierarchical regression analysis for variables predicting WM performance in the Selective updating composite score at the second assessment point between March 23 and April 07, 2020. | | | | | | | | | | | | | | |
| --- | --- | --- | --- | --- | --- | --- | --- | --- | --- | --- | --- | --- | --- | --- |
|  | Step 1 | | | |  | Step 2 | | | |  | Step 3 | | | |
|  |  | | | |  |  | | | |  |  | | | |
| Predictor | *B* | *SE B* | β | Sig. |  | *B* | *SE B* | β | Sig. |  | *B* | *SE B* | β | Sig. |
| Age | -0.02 | 0.01 | -0.172 | 0.012 |  | -0.02 | 0.01 | -0.188 | 0.007 |  | -0.02 | 0.01 | -0.189 | 0.007 |
| Gender | -0.10 | 0.12 | -0.059 | 0.389 |  | -0.11 | 0.12 | -0.063 | 0.36 |  | -0.11 | 0.12 | -0.063 | 0.365 |
| Education | 0.04 | 0.02 | 0.146 | 0.033 |  | 0.04 | 0.02 | 0.145 | 0.034 |  | 0.04 | 0.02 | 0.145 | 0.034 |
| Openness | 0.00 | 0.01 | -0.011 | 0.873 |  | 0.00 | 0.01 | -0.009 | 0.889 |  | 0.00 | 0.01 | -0.010 | 0.882 |
| Conscientiousness | 0.01 | 0.01 | 0.113 | 0.107 |  | 0.01 | 0.01 | 0.123 | 0.082 |  | 0.01 | 0.01 | 0.122 | 0.085 |
| Trait anxiety | -0.01 | 0.01 | -0.079 | 0.265 |  | -0.01 | 0.01 | -0.134 | 0.103 |  | -0.01 | 0.01 | -0.135 | 0.105 |
| Reasoning | 0.10 | 0.02 | 0.405 | < .001 |  | 0.10 | 0.02 | 0.405 | < .001 |  | 0.10 | 0.02 | 0.405 | < .001 |
| State anxiety time 2 |  |  |  |  |  | 0.03 | 0.02 | 0.102 | 0.186 |  | 0.03 | 0.02 | 0.101 | 0.211 |
| COVID-19 anxiety time 2 |  |  |  |  |  |  |  |  |  |  | 0.00 | 0.03 | 0.005 | 0.941 |
| *R^2^* |  | 0.197*** |  |  |  |  | 0.204*** |  |  |  |  | 0.204*** |  |  |
| *R^2^* change |  |  |  |  |  |  | < .001 |  |  |  |  | < .001 |  |  |
| * indicates *p* < .05 ** indicates *p* < .01 *** indicates *p* < .001 | | | | | | | | | | | | | | |
